# Realizing ambitions: A framework for iteratively assessing and communicating national decarbonization progress

**DOI:** 10.1016/j.isci.2021.103695

**Published:** 2021-12-25

**Authors:** Chuan Zhang, Honghua Yang, Yunlong Zhao, Linwei Ma, Eric D. Larson, Chris Greig

**Affiliations:** 1 Energy Systems Analysis Group, Andlinger Center for Energy and the Environment, Princeton University, Princeton, NJ 08544, USA; 2 State Key Laboratory of Power Systems, Department of Energy and Power Engineering, Tsinghua University, Beijing 100084, China

**Keywords:** Energy resources, Energy policy, Energy systems

## Abstract

A growing number of governments are pledging to achieve net-zero greenhouse gas emissions by mid-century. Despite such ambitions, realized emissions reductions continue to fall alarmingly short of modeled energy transition pathways for achieving net-zero. This gap is largely a result of the difficulty of realistically modeling all the techno-economic and sociopolitical capabilities that are required to deliver actual emissions reductions. This limitation of models suggests the need for an energy-systems analytical framework that goes well beyond energy-system modeling in order to close the gap between ambition and reality. Toward that end, we propose the Emissions-Sustainability-Governance-Operation (ESGO) framework for structured assessment and transparent communication of national capabilities and realization. We illustrate the critical role of energy modeling in ESGO using recent net-zero modeling studies for the world's two largest emitters, China and the United States. This illustration leads to recommendations for improvements to energy-system modeling to enable more productive ESGO implementation.

## Graphical abstract

**Figure:**
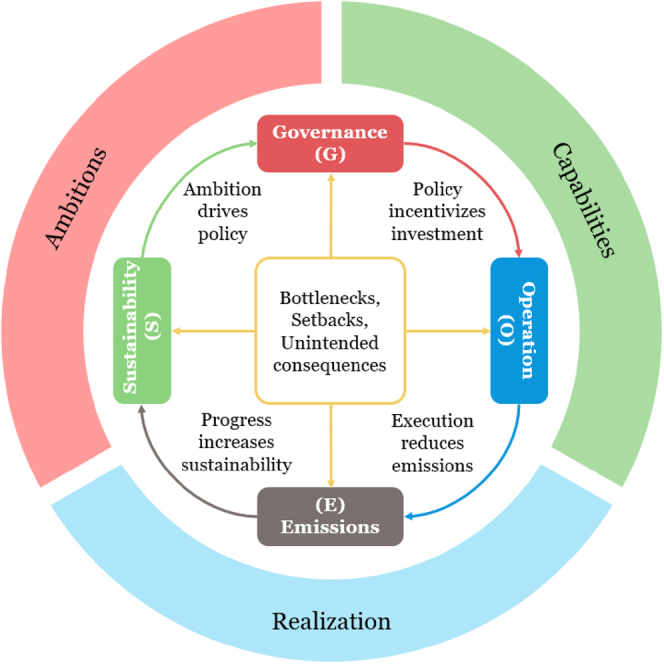

## Introduction and literature review

The Paris Agreement signaled the start of a new era for the climate mitigation agenda (UN, 2015). An increasing number of countries are now ratcheting up their ambitions for greenhouse gas (GHG) emissions reductions, including China with its pledge of carbon neutrality no later than 2060 (Xinhua, 2020) and the United States with a pledge of net-zero emissions by 2050 (the White House, 2021a), among others. *Ambition* is necessary, but not sufficient for a nation to achieve deep decarbonization of its economy. Success also depends on a country's *capabilities* to deliver on the ambition. Measures of capability include natural resource endowments (e.g., solar insolation or geologic CO_2_ storage capacity), industrial manufacturing capabilities, financial strength, human capacity, and societal and political circumstances (e.g., institutions, governance mechanisms, and market designs). Moreover, the pace of decarbonization is ultimately decided by *realization*, i.e., the leveraging of capabilities to mobilize physical capital (energy efficiency, low-carbon power plants, factories, buildings, and vehicles, and enabling infrastructure like high-voltage transmission lines and CO_2_ storage resources) and social capital (laws and regulations, effective energy markets, and community support) to deliver emissions reduction ambitions.

There are many published global and national deep decarbonization pathways (IPCC, 2014; IDDRI, 2017; IPCC, 2018; Energy Foundation China, 2020; IEA, 2021), including, recently, a range of 1.5°C-compatible pathways for China emanating from different integrated assessment models (Duan et al., 2021). Most of such previous studies have used either techno-economic models, macroeconomic models, or a combination of them to propose future energy scenarios and technological pathways, essentially answering “what-if” questions, such as “what will the modeled energy system in a future year look like if a particular technology option is deployed twice as rapidly as it is currently being deployed?”, but assessing the feasibility of achieving the resulting pathways in only limited ways, if at all (Gambhir, 2019; Pye et al., 2021). As a result, emissions trajectories for most countries have lagged the progress anticipated in modeling, resulting in a large gap between actual GHG emission reductions and those necessary to satisfy the Paris Agreement (Luderer et al., 2018; UNEP, 2019).

Feasibility of modeled pathways depends on capabilities within a region and their effective deployment. We posit that inadequate consideration of capabilities and their mobilization has resulted in modeling outcomes that are not being translated into effective enough policies and social actions. Or the other way around, effective energy modeling expertize to inform successful climate policy has not been established (Dioha et al., 2020; Pye et al., 2021; Rogelj et al., 2021). In the real world, energy decision making at national or local levels is a complex function of political, technological, social, environmental, and economic contexts. Current energy-systems modeling doesn't generally capture one or more elements of this complexity. For example, Ford and Abdulla (2021) identify investments in energy infrastructure as facing different risk levels, and thus national readiness for deploying complex energy infrastructure needs to be evaluated, necessitating a change in development paradigm; Capros et al. (2019) call for energy-system modeling to take into account ambitions, policy feasibility, technological uncertainty, and required capability building in order to best inform EU strategy toward climate neutrality. Geels et al. (2017) propose a “sociotechnical” conceptual framework to address the multidimensional challenges of deep decarbonization with focus on the bottom-up driving force of niche innovations. In addition, whereas governance ambition is crucial for guiding the transition to a climate-neutral energy system (Millot et al., 2020), some studies consider emissions reduction ambitions for certain sectors, but not national net-zero emission ambitions (Löffler et al., 2019; Yang et al., 2021), and others target national emissions reductions that are less than announced ambitions (He et al., 2016). Most modeling studies produce decarbonization pathways using some form of least-cost optimization, but lack feasibility analysis such as the availability of resources required by these paths and social acceptance. Examples of such studies include ones for China (Li et al., 2020; Zhang and Chen, 2021; Song et al., 2022), for the U.S. (Denholm et al., 2021; Cole et al., 2021), and for the E.U. (Millot et al., 2020; Löffler et al., 2019). Some studies have done well in examining natural resource endowments (Liu et al., 2020), but have not assessed the capability gap to utilize these resources. For example, Denholm et al. (2021) did not assess the availability of needed raw materials, Li et al. (2022) did not consider manufacturing capabilities needed to exploit natural resource endowments, and Bednar et al. (2021) failed to consider financial investment gaps. Another shortcoming in existing studies comes from insufficient granularity for understanding prospects for realization, such as localized site identification and suitability assessments for energy infrastructure considering physical landscape features and local community support or opposition (Iyer et al., 2017; Dominković et al., 2016). Higher granularity would provide more actionable information for decision-makers (Bistline 2021), thus helping to bridge the gap between existing policies and long-term pathways.

Our review of the literature points to the need for improved energy-systems modeling in at least four main aspects. Firstly, the granularity of energy models needs to be increased to more effectively reflect: the temporal and spatial organization of energy supply and use, the demand response to a changing energy system, the impact of extreme climate-related events, and other factors (Azevedo et al., 2021; Shove, 2021). Secondly, energy models need to open boundaries to facilitate interdisciplinary modeling such as coupling energy models with material flow and other sector models (Kullmann et al., 2021). Thirdly, model results need to be reported in a transparent and comprehensible manner, which is also the key to link technical modeling and practical implementation (Aryanpur et al., 2019; Amer et al., 2020). For example, assumptions, constraints, and inputs and outputs must be transparently specified so that limitations in modeling results are also clear (Vaidyanathan, 2021). Open-source databases and open platforms for energy modelers would also improve transparency and cross-model fertilization (Morrison, 2018; Lerede et al., 2021). Fourthly, differences in the capabilities between individual countries or regional groupings of countries are not adequately compared in modeling communities (Schreyer et al., 2020; Fragkos et al., 2021), missing important and nuanced challenges, opportunities and synergies across countries, which impact on pathway feasibility. Moreover, differences in the thinking patterns of modelers in different countries are also reflected in the modeling process and interpretation of the model results (Haikola et al., 2021). Understanding and appreciating these differences is essential for international communication and cooperation on global climate governance.

In summary, modeling energy-system transitions to net-zero emissions require considerations in multiple dimensions that are difficult to capture in any single modeling exercise, and this helps explain why model outcomes generally have not been translated into effective policies and social actions. Taken together, the limitations in energy-system modeling suggest the need for an energy-systems conceptual analysis framework that goes beyond energy-system modeling itself (Wiese et al., 2018; Chang et al., 2021)—one that blends techno-economic optimization, macro-economic modeling, business analysis, stakeholder engagement, expert consultation, international comparison, and other methods to help accelerate the translation of ambition into progress toward net-zero emissions globally. Toward that end, we propose here a common framework for structured assessment and transparent communication of national capabilities and realization. This framework is designed to help:

(a) Guide the design of more-effective policies for the transition to net-zero emissions in individual countries.
(b) Identify challenges to the transition that are common across countries and that might be more effectively addressed cooperatively.
(c) Identify opportunities for synergistic collaboration between countries with different but complementary capabilities.

The next section introduces our proposed Emissions-Sustainability-Governance-Operations (ESGO) analytical framework. We then illustrate the application of this framework to link the role of national ambitions, capabilities, and realization across six essential pillars needed to support transitions to net-zero emissions in any economy: 1) end-use efficiency and electrification, 2) clean electricity supply, 3) clean fuels supply, 4) CO_2_ capture, utilization and storage, 5) reduced non-CO_2_ emissions, and 6) enhanced land sinks (natural carbon solutions). For this illustration, we compare results of highly regarded and influential low-carbon transition studies for the world's two largest emitting countries completed with involvement of the authors, one for China (He et al., 2020; ICCSD, 2021), and one for the United States (Larson et al., 2021). This comparative analysis provides a means for understanding and appreciating the considerable differences between the two countries' capabilities and for translating, through application of the ESGO framework, capabilities into effective strategies for realizing ambitions. In our discussion section, we reflect on improvements needed in energy-systems models to maximize their utility in the ESGO process, as well as on how the ESGO process itself can inform what model improvements are needed.

## Linking ambition, capability, and realization

Historically, national energy transitions have involved ever-increasing resource consumption to meet energy service demands, with limited attention to environmental and social values. In recent decades, this led to sustainability crises that threatened the ongoing license to operate the then-current energy systems. Regulators, consumers, and communities reacted to effect changes to address the crises. For example, horrendous pollution from thermal coal plants in the U.K. in the early/mid-1900s led to the closure of coal mines, and the economic disruption from three global oil supply crises since the early 1970s led many countries to implement energy diversification, efficiency, and other strategies to increase energy independence. The current energy transition to address climate change is motivated almost entirely by ambitions to make energy services environmentally and socially sustainable. Governance structures are increasingly aligning with this emerging ambition, but have thus far failed to achieve desired outcomes, i.e., deep reductions in GHG emissions consistent with Paris goals.

**Figure 1:**
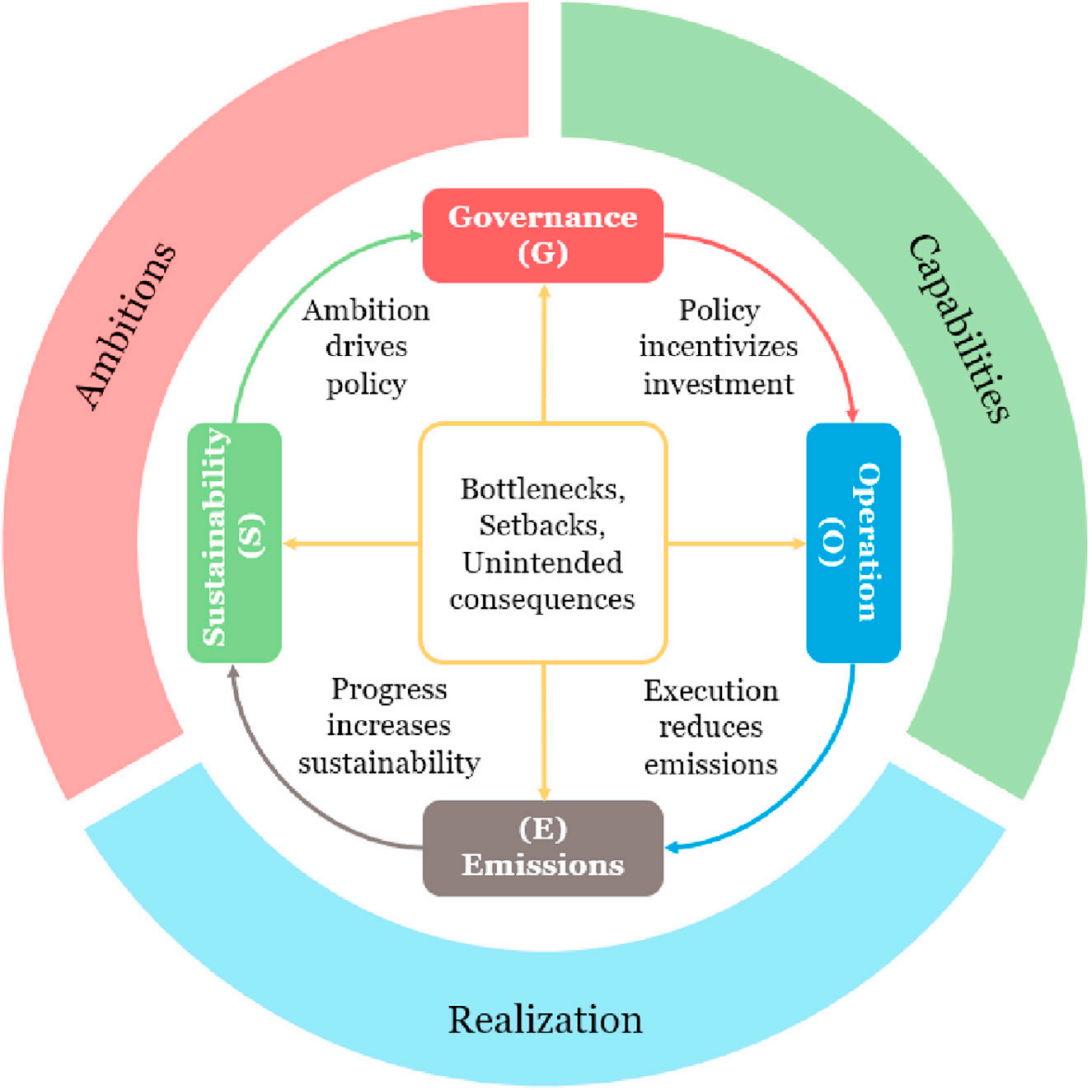
The Emissions-Sustainability-Governance-Operations (ESGO) framework for accelerating national decarbonization processes through elevating ambition, identifying and mobilizing capability, and designing policy to advance realization

To understand this gap between mitigation, ambition, and emissions reductions outcomes, we explore the interactions between ambition, capabilities, and realization in energy transitions. ESGO is a framework for translating the ambition of a net-zero emissions future into enabling policies that incentivize the necessary investments to leverage local capabilities and effectively enable the realization of material GHG emissions reductions. Our framework is illustrated in Figure 1. The relationships linking ambition and capability to realization are described though the frame of emissions, sustainability, governance and operation, building on ideas of Ma, et al. (2021). The ESGO framing acknowledges the imperative that achieving net-zero carbon emissions is essential for sustainable development, while governance and operation (of markets and physical assets) are critical for driving emissions reductions via physical changes. Bottlenecks, setbacks, and unintended consequences may emerge and act to slow the pace of transitions, but leveraging the four connected elements of ESGO to continuously identify and resolve those barriers can lead to ambition, capabilities, and realization becoming mutually reinforcing and accelerate the process of decarbonization. Systematic and iterative application of the ESGO framework, encompassing energy-system modeling, localized case studies, stakeholder engagement, documentation of lessons learned, and respecting the requirements of local political and international governance institutions, will help assess and communicate net-zero emission pathways to 1) elevate ambitions, 2) identify and mobilize capabilities, and 3) design policies and interventions that advance realization. We note that while energy-system modeling is an essential input within the ESGO framework, it is only one element involved in the process that the framework represents.

### Elevating ambition

Ambition here refers to the level of ambition for tackling climate change in national governance, which is a function of external and internal influences. External influences are reflected in, for example, a national government's Paris Commitments. These are typically announced to international communities. The level of ambition may be decided based on a nation's historical contributions to global GHG emissions, its perceived vulnerability to impacts from climate change, its international positioning and strategy, and/or other factors. External levels of ambition may be influenced by a country's

• Current and expected future demands for energy services and dependence on fossil fuels;
• Economic and energy independence/dependence on other entities;
• Global standing in production, finance, military, knowledge, and other dimensions;
• Influence from other nations and geopolitical institutions.

Internal influences stem from the desires and demands from society within a nation to tackle climate change. These can be observed from the attitudes and determination of various stakeholders within a nation toward climate change. Internal ambition might result from the diverse goals, activities, and influence of different groups within society, such as environmental non-profits, activists, youth movements, incumbent industrial firms, and new-energy and climate-sector entrants. Internal ambitions may be influenced by

• Political structures and decision processes (top-down, bottom-up, consensus, etc.);
• Level of majority/control/tenure of incumbent political leadership;
• Economic and social structure, such as who is more active and powerful in economic development and social progress;
• A country's moral and cultural characteristics, education level, and other factors.

To assess and communicate a nation's net-zero emission pathway, it is first necessary for a government to establish and elevate suitable emissions targets, based on both internal and external drivers of ambition. The targets not only help stimulate global ambition to deal with climate change but also inspire and guide major stakeholders within the nation.

### Identifying and mobilizing capabilities

Here, capabilities refer to the characteristics and endowments that a country has to facilitate a transition to net-zero emissions. We distinguish between techno-economic, sociopolitical, and coupled capabilities.

Techno-economic capabilities mainly refer to indigenous resources and capabilities to deploy and adapt low-carbon technology and infrastructure and pursue technological innovation in support of net-zero emissions. These include, but are not limited to:

• Natural resource endowments and development capacity that enable the exploitation of low-carbon energy resources and natural carbon solutions (enhancing land sinks);
• Capacity and condition of energy conversion facilities, energy infrastructure, and energy utilization facilities (buildings, factories, vehicles, etc.);
• The current and prospective effectiveness of markets in supporting decarbonization;
• Capacity of industrial systems—engineering, supply chains, manufacturing, and project delivery;
• Capacity to mobilize risk-capital for project development.

Sociopolitical capabilities refer to institutional and human capacities for social reform, strategic planning, and policy making and implementation. Compared to the techno-economic capabilities, most sociopolitical capabilities are even less well considered or represented in current energy-system modeling and policy analysis (Peng et al., 2021). Socio-political capabilities include, but are not limited to:

• The relative influence and decision-making authority of entities and individuals, in both government and civil society, engaged in emissions reduction;
• Current public policies (legislation and regulation) supporting a net-zero transition;
• Governmental capacity for implementing and enforcing new laws and regulations aimed at realizing net-zero emissions;
• Technological and societal roadmaps, whether produced by governmental or non-governmental organizations, for realizing net-zero emissions;
• Effectiveness of education programs and public advertising campaigns in informing and encouraging low-carbon consumer behaviors;
• Level of public trust in institutions;
• Capacity of workers, managers, and other human resources needed to execute the transition.

Coupled capabilities mainly refers to the strength of innovation and capacity-building ecosystems, which draw on techno-economic and sociopolitical capabilities to more effectively build and enhance needed capabilities.

### Designing policies and interventions that advance realization

The existence of capabilities does not by itself guarantee that ambitions for net-zero emissions will be realized in a timely fashion or even at all. Rapid (e.g., by mid-century) realization of a net-zero ambition requires immediate actions (starting today) that leverage existing capabilities to realize near-term ambitions and that invest in incubating and establishing new capabilities for realizing the longer-term ambition. The readiness of a nation to realize its ambitions depends on the ability of the society to act quickly to deploy existing technologies, to innovate to improve technologies, and to mobilize capital to deploy technologies at the scale needed for a net-zero emissions economy. Within the ESGO framework key actions to advance realization include, but are not limited to:

• Identifying and monitoring domestic GHG emission sources and climate impacts;
• Creating strong and enduring social commitments to the net zero-emissions ambition across the whole of society;
• Engaging with international communities to create a strong and enduring global commitment and cooperation including transition-enabling trade;
• Forming, promulgatin,g and ratcheting up effective policies which motivate investment in the development/deployment of natural resources, technology, and infrastructure needed to progressively decrease emissions in accordance with the ambition;
• Recognizing that uncertainties around the long-term feasibility of certain pathways requires investing to expand the portfolio of mitigation options to provide the capacity to pivot to alternative pathways in the event setbacks and bottlenecks emerge to thwart progress toward long-term realization.

We acknowledge that the ambition, capability, and realization taxonomy may not cover all dimensions of national energy transitions, but insights from adopting these perspectives can help clarify key questions in net-zero energy transitions, including but not limited to: the pace and profile of the planned emissions reduction trajectory, the scope of greenhouse gases considered (CO_2_ only or all GHGs), and the relative contributions of GHG reductions, removals, and offsets. ESGO offers a methodological framework to translate a county's ambition/capability/realization positioning into an executable plan. Iterative review and updates of the ambition, capabilities, and realization are essential to applying the ESGO framework so as to be responsive to past performance, along with shifting political priorities, social attitudes, and international influences.

## National net-zero modeling case studies

Here, we demonstrate how national net-zero emissions pathway modeling studies, in the context of the ESGO framework, can help elucidate differences in *ambition* and *capabilities* across different countries, and implications for *realization* of ambitions. To be effective, the ESGO framework requires inputs from energy-system transition modeling carried out with much greater sectoral, temporal, and spatial resolution than has been typical in past studies. We illustrate how more-granular modeling of low-carbon pathways can inform cross-country comparative assessments of low-carbon ambitions, and capabilities for realizing these, by comparing results from major low-carbon transition studies for China and the United States. We start with brief overviews and comparative summaries of these studies. The supplemental information (SI) document gives additional details.

### Overview of the China and U.S. studies

China's Long-term Low-Carbon Development Strategy and Pathway (CLLDSP) study (He et al., 2020), led by the Institute of Climate Change and Sustainable Development (ICCSD) at Tsinghua University, organized 24 top-level research institutes to carry out 18 subprojects covering and integrating a wide range of key issues (see Figure S1). Initially, the aim of the project was mainly to support China's reporting of its 2050 strategy to the UNFCCC; it ultimately also provided key inputs for China's announcement of 2060 carbon neutrality (CGTN, 2020). The project adopted a comprehensive methodology to maximize the combined understanding and insights offered through modeling, data analysis, and expert knowledge. In addition to using holistic and sub-domain models for data integration, 15 workshops were held to promote cross-cutting analysis and interdisciplinary exchange among the participating institutions. This allowed the project to benefit from a combination of qualitative and quantitative analysis, and across the physical and social sciences.

**Figure 2:**
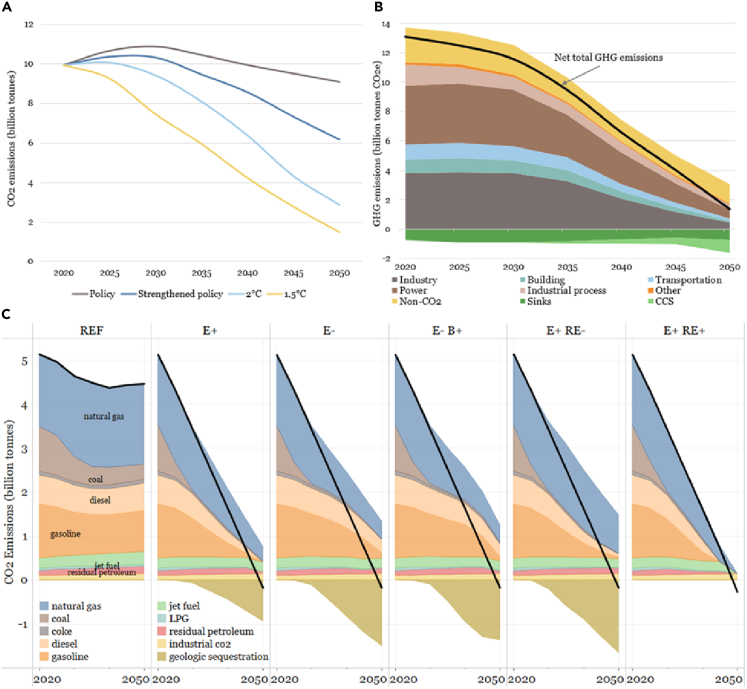
Decarbonization scenarios in the China and U.S. modeling studies (A) Energy-related CO_2_ emissions of four investigated emission trajectories for China (He et al., 2020). (B) All GHG emissions for the 1.5°C scenario for China (He et al., 2020). (C) CO_2_ emissions from the U.S. energy/industrial system for five pathways achieving net-zero emissions economy wide by 2050 and one reference pathway without CO_2_ mitigation ambitions (Larson et al., 2021). Black lines show net energy/industrial emissions. See supplemental information for definitions of the pathways shown here.

Four emission scenarios were initially proposed and simulated (Figure 2A), including Policy scenario, Strengthened Policy scenario, 2°C scenario, and 1.5°C scenario. Among these, the 1.5°C scenario (Figure 2B) incorporates, as judged by the study authors, all possible efforts to achieve the lowest emissions by 2050. Within the ESGO framework, this work can be understood and communicated conceptually as follows:

1. *Elevate ambitions:* Two simultaneous ambitions were proposed and guided the outputs of all subprojects. One, driven by internal influences, is to ensure sustained economic growth so as to achieve the goal of becoming a socialist modern power by 2050. The second, driven by external influences, is to strive to support realization of the Paris Agreement goals of less than 2°C or 1.5°C global warming. These co-ambitions reveal the core challenge for China's net-zero emission pathways: how to ensure economic growth while controlling emissions.
2. *Identify and mobilize capabilities:* The scenario calculation is mainly based on the research results of more granular subprojects in four sectors: power, transportation, industry, and buildings. These subprojects were led by authoritative experts in relevant fields to ensure that the techno-economic capabilities to achieve net-zero emissions can be defined. The 1.5°C scenario (Figure 2B) reflects the emission reductions that these four sectors can achieve to the best of their techno-economic capabilities. At the same time, other subprojects are mainly used to analyze the required support and comprehensively integrate these scenarios. Even so, after communication between the project team and government departments, it was found that there is still a lack of capabilities to immediately reduce total emissions as required by the 2°C or 1.5°C scenario (Figure 2A). Therefore, the final tradeoff generated two additional scenarios, including the Low-Carbon Transition (LCT) Scenario and 2050 Net-Zero CO_2_ Emission (NZ2050) scenario (see Figure S2). These firstly reach the carbon peak before 2030 according to the Strengthened Policy scenario, and then accelerate emission reductions to realize near-zero emissions by 2050. This also verifies our understanding that it is difficult to fully consider all capabilities required to achieve net-zero emissions only by relying on current energy models, especially sociopolitical capabilities which are more often neglected.
3. *Design policies and interventions that advance realization:* Because techno-economic capabilities have been considered in the scenarios, the final policy proposals offered by the study team are mainly aimed at measures to strengthen sociopolitical capabilities. Specific proposals for the short term (2021–2025) are largely reflected now in China's 14^th^ Five-Year Plan. Broader proposals for the longer term are reflected in the announcement of China's strengthened 2030 carbon-peak targets and its 2060 carbon neutrality target. Policies for capability-building that bridge the short-term actions and long-term ambition are still lacking, however, especially for enhancing sociopolitical capabilities. Driven by this problem, on 26 October 2021, the State Council of China issued “Working Guidance for Carbon Dioxide Peaking and Carbon Neutrality in Full and Faithful Implementation of the New Development Philosophy” (Xinhua, 2021), signaling that China has begun to comprehensively build the policy system required to achieve net-zero emissions.

The Net-Zero America (NZA) study (Larson et al., 2021), led by researchers at Princeton University and involving a large team of researchers across several other U.S. organizations, aimed to provide actionable analysis to inform public and private decision making at national, state, and local levels. Using a suite of models (see Figure S2), the NZA study created multiple pathways by which the U.S. energy and industrial system could contribute to achieving net-zero GHG emissions economy wide by 2050, factoring in expert understanding about future non-CO_2_ emissions and enhanced land sinks. Each energy/industrial pathway meets an exogenously imposed linear reduction in CO_2_ emissions to 2050 (Figure 2C). By varying other exogenous constraints, five different technological pathways were quantified at a relatively coarse (14-region) geospatial resolution. Various modeling methods were then applied to “downscale” those results to state, county, or finer spatial resolution, enabling stakeholders to more clearly grasp the implications and impact of pathways to net-zero emissions. Within the ESGO framework, the NZA study can be understood and communicated conceptually as follows:

1. *Establish ambition:* The NZA study considered the ambition of net-zero greenhouse gas emissions by 2050 for the country in the context of growth in energy services from 2020 to 2050 consistent with annual economic growth of 1.9% per year.
2. *Provide insight relating to capabilities*: By constraining different key modeling input variables, five distinct pathways to net-zero were created. For example, in one pathway, a cap was imposed on the annual amount of wind and solar generating capacity that could be deployed, and in another, the rate at which the transportation sector electrifies was constrained. The differentially constrained pathways provide insights into the capabilities that would need to be deployed to achieve net-zero under different possible future conditions. The feasibility of achieving any particular pathway was not explicitly assessed in the work but can be gauged to a certain degree by comparing the pace of change needed along a given pathway against the pace at which analogous changes have been achieved historically. Gaps between historical experience and modeled pathways point to where increased capabilities are needed to achieve ambitions.
3. *Inform policies and actions to advance realization*: The NZA modeling quantified targets for technology deployments over time, for which policies must be designed to ensure that both techno-economic and sociopolitical capabilities for achieving the targets are developed and deployed. Legislation passed by the 117^th^ U.S. Congress and signed into law by President Biden (the White House, 2021b) in 2021, together with the pending (as of this writing) “Build Back Better” legislation (the White House, 2021c) reflect findings and insights from the NZA study, including specific common actions in the 2020s that were found to be needed across all five NZA pathways, for example expansion of solar and wind generation and electrification of the light-duty vehicle fleet to greater or lesser degrees. What policies will be needed in the long-term future to ultimately achieve the 2050 net-zero goal will depend on the extent of progress made in the next few years. New modeling studies in the future that take into consideration the progress made (or lack thereof) to that point in time can then help inform what future capabilities are needed and policies can be formulated to target those needs. This would represent iterative application of the ESGO framework.

### Comparing results of the China and U.S. modeling studies

The China and U.S. studies differ in their approaches, but both rely on the same six key pillars of decarbonization:

1. End-use energy efficiency and fuel switching to electricity (electrification), especially in the transportation and buildings sectors.
2. Clean (low, zero, or negative carbon) electricity generation, including from variable renewables (wind and solar) and firm sources to balance the variable resources (biomass, nuclear, gas with CO_2_ capture and storage, batteries), as well as expanded transmission to move remotely generated electricity to demand centers.
3. Clean (zero or negative-carbon) liquid and gaseous fuels, including biofuels, hydrogen, and synthesized liquid hydrocarbons.
4. CO_2_ capture and utilization or storage (CCUS).
5. Reduced emissions of methane and other non-CO_2_ GHG gases.
6. Enhanced uptake of atmospheric CO_2_ in the biogeosphere (i.e., nature-based solutions), providing negative emissions.

A comparative delving into each of these pillars for China and the U.S. surfaces similarities and differences in decarbonization ambitions and sheds light on capabilities that might be brought to bear in pursuit of the ambitions. We select for these illustrative comparisons the 1.5°C pathway from the China study and the E+ pathway from the U.S. study (see supplemental information for definitions of these pathways). Table 1 provides a high-level comparative summary across all six pillars.

**Table 1:** National comparisons for six decarbonization pillars from the China and U.S. modeling studies

	China	US
Pillar 1: Improve energy productivity through efficiency and electrification	Primary energy use grows from 145 EJ in 2020 to 155 EJ in 2030 and then declines to 146 EJ in 2050. Energy intensity falls by 3.5%/y from 2020 to 2030 and 4.5%/y from 2030 to 2050.	Primary energy use falls by 30%, from 98 EJ in 2020 to 70 EJ in 2050 and energy intensity declines an average of 3.1%/y during this period.
	Electricity as a fraction of final energy use increases from 1/5 today to 2/3 in 2050.	Electricity as a fraction of final energy use increases from 1/5 today to 1/2 in 2050.
Pillar 2: Clean electricity	The portion of solar and wind electricity generation reaches 60% in 2050.	The portion of solar and wind electricity generation reaches 85% in 2050.
	Nuclear, hydro, coal with CCS, gas with CCS, and biomass account for 16%, 10%, 6%, 3%, and 2% of total generation in 2050, respectively.	Nuclear, hydro, gas with CCS, gas without CCS, and biomass account for 5%, 3%, 2%, 2%, and 1% of total generation in 2050, respectively.
	157 GW of coal capacity with CCS in 2050.	87 GW of gas capacity with CCS in 2050.
Pillar 3: Clean fuel	Hydrogen production (from all sources) is 60 million t/y in 2050.	Hydrogen production (from all sources) is 58 million t/y in 2050.
	Potential primary biomass supply for energy is estimated to be 9 to 15 EJ/y by 2050.	Potential biomass supply for energy is 13 EJ/y by 2050, all of which is utilized.
Pillar 4: CCUS	1 Gt CO_2_/y captured and stored by 2050.	1 Gt CO_2_/y captured and stored by 2050.
Pillar 5: Non-CO_2_ GHGs	2.38 GtCO_2e_ in 2020; 1.2 GtCO2e in 2050.	1.25 GtCO_2e_ in 2020; 1 GtCO_2e_ in 2050.
Pillar 6: Land Sink	Increases from 0.7Gt CO_2_ in 2020 to 0.8 GtCO_2_ in 2050 (a peak of 0.9 Gt in 2030).	Increases from 0.7Gt CO_2_ in 2020 to 0.85 GtCO_2_ in 2050.

Sources (He et al., 2020; Larson et al., 2021).

#### Pillar 1

**Figure 3:**
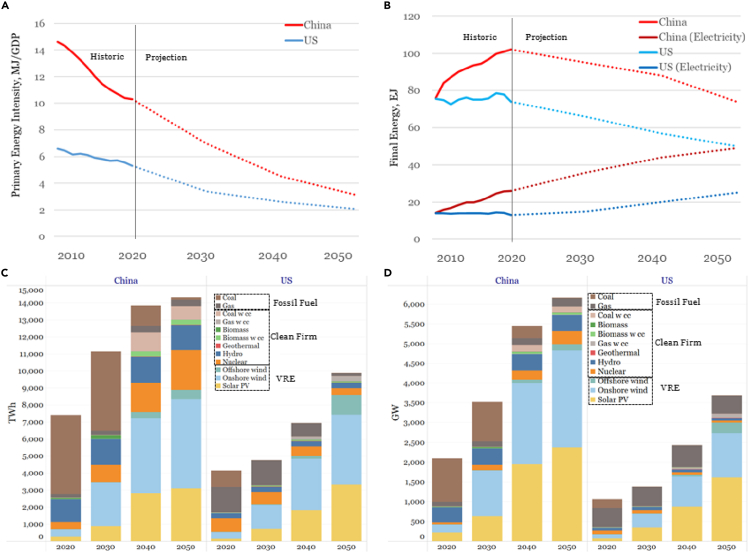
Projections of energy intensity, energy use, electricity generation, and capacity for U.S. and Chinese decarbonization scenarios (A) Historic and projected primary energy intensity (MJ per $GDP). (B) Historic and projected final energy use (total and electricity portion). (C and D) Projected electricity generation; and (D) projected installed electricity generating capacity. Panels (C) and (D) distinguish variable renewables (solar and wind), clean firm (hydro, nuclear, biomass, geothermal, fossil with CO_2_ capture), and fossil fuels (without CO_2_ capture). Sources (He et al., 2020; Larson et al., 2021).

Ambitious improvements in energy efficiency (Figure 3A) and electrification (Figure 3B) characterize both the U.S. and China decarbonization pathways. The declines in energy intensity result from assumed decreases in energy use per unit of energy service delivered and shifts in economic structures toward higher value-added activities, as well as increased electrification of industrial production and massive expansion of both heat-pump electric heating of buildings and battery-electric light-duty vehicles.

#### Pillar 2

Both the U.S. and China studies include ambitious goals for expanding electricity generation and the portion from carbon-free generation, both variable renewables (e.g., solar and wind) and clean firm generation (hydro, nuclear, biomass, geothermal, fossil with CO_2_ capture).

#### Pillar 3

Despite the rapid electrification in both the U.S. and China low-carbon scenarios, end-use demands for fuels and feedstocks are still around 25 EJ in 2050 in both studies. The U.S. study details how these fuel demands are satisfied: about 65% are met using fossil fuels whose emissions are offset by negative emissions elsewhere in the economy; hydrogen provides about 20%; and the rest are in the form of lignocellulosic biofuels and fuels synthesized from hydrogen and captured CO_2_. The fuel sector in the China study was not modeled in detail, but hydrogen is expected to play an important role, accounting for about 10% of end-use energy.

#### Pillar 4

**Figure 4:**
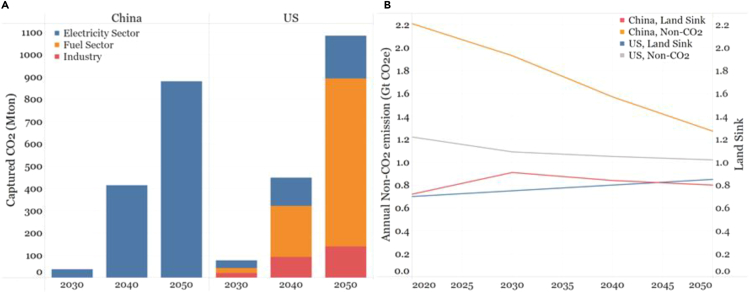
Comparison of modeled CO_2_ capture and non-CO_2_ emission between U.S. and China for net-zero (A) Annual source of captured CO_2_ for U.S. and China. (B) Annual non-CO_2_ greenhouse gas emissions and land-based greenhouse gas sink for U.S. and China. Sources (He et al., 2020; Larson et al., 2021).

An ambitious CO_2_ capture and storage rate of close to 1 billion t/year in 2050 are assumed in both the China and U.S. studies. Most (72%) of the CO_2_ capture in the U.S. study occurs at hydrogen and fuels production facilities, with the rest split between power sector and cement plants. In the China study, coal continues to play an important role in power generation, necessitating that most of the CO_2_ capture occurs in the power sector (Figure 4A).

#### Pillar 5

Non-CO_2_ GHG emissions decline to 1 GtCO_2e_ in 2050 in the U.S. analysis and 1.2 GtCO_2e_ in the China analysis (Figure 4B). Both studies concluded that further reductions, regardless of cost, will be difficult to achieve with today's level of understanding of mitigation options.

#### Pillar 6

Land carbon sinks (i.e., annual removal of carbon from the air and permanent storage in soil or trees) are critical for net-zero emission scenarios because they offset positive greenhouse gas emissions from elsewhere in the economy. The U.S. study assumed the land sink grows from 0.7 Gt in 2020 to 0.85 Gt in 2050, whereas in the China study it increases from 0.7Gt in 2020 to 0.8 Gt in 2050 with a peak of 0.9 Gt in 2030 (Figure 4B).

### Modeling that helps assess decarbonization capabilities and benchmark progress

**Figure 5:**
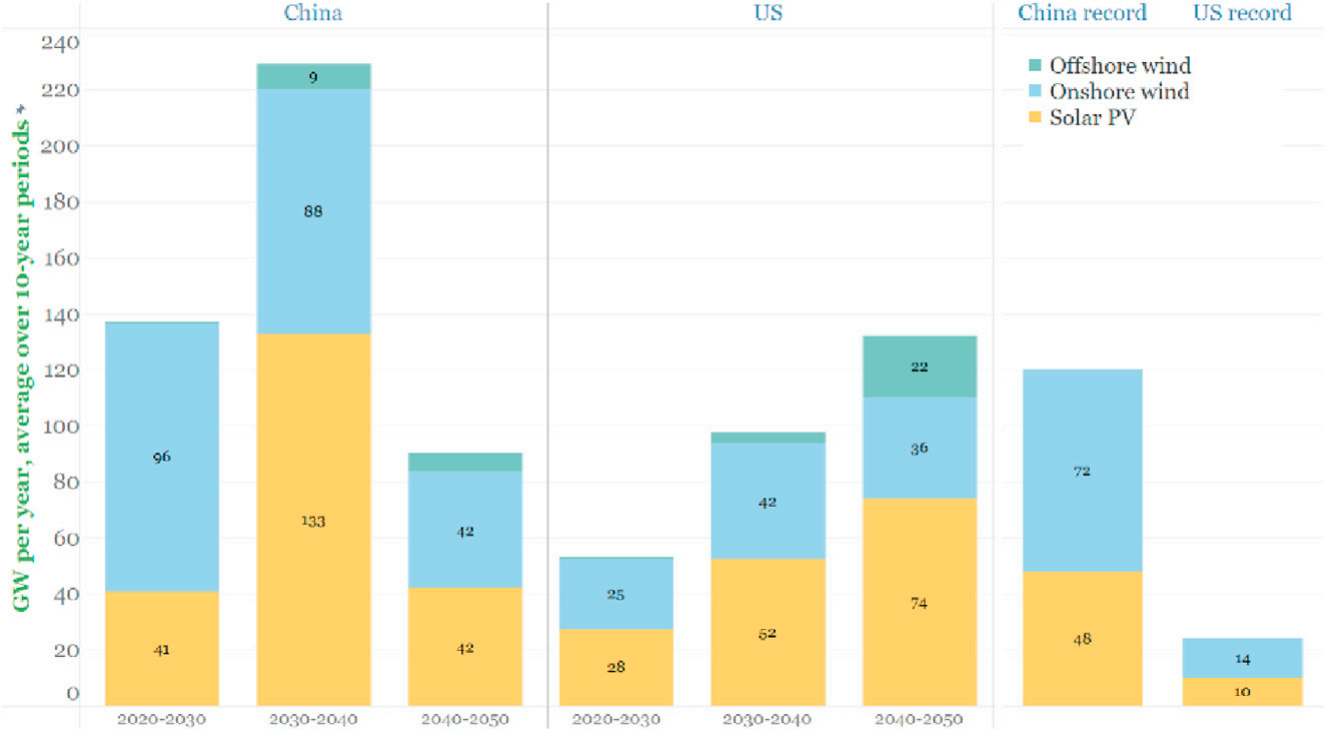
Average annual expansion (GW/year) each decade in solar and wind generating capacity in the Chinese and U.S. decarbonization studies The two rightmost bars show the largest single-year expansion in wind plus solar ever achieved, which occurred in 2020 (He et al., 2020; IRENA, 2021; Larson et al., 2021).

Quantifying the changes needed in a country to achieve decarbonization ambitions, as above for China and the U.S., provides one way to gauge the capabilities needed in a country for realizing its ambitions. Comparisons between the capabilities required in decarbonization pathways and historically demonstrated capabilities offers one indication of the feasibility of achieving ambitions. As one example, consider the levels of solar and wind electricity generation envisioned in the two studies (Figures 3C and 3D). The corresponding solar and wind capacity that would need to be added each year to deliver the envisioned electricity are shown in Figure 5. The two rightmost bars in the figure show the maximum amount of capacity started up in any previous year, which in both countries was 2020. China installed a record 72 GW of wind and a near-record 48 GW of solar PV that year. This combined 120 GW of new capacity comes close to the average build rate in the 2020s envisioned in China's decarbonization scenario, but the rate would need to be nearly double this in the 2030s. The U.S. started up 24 GW of wind plus utility-scale solar PV in 2020 (plus about 5 GW of rooftop solar). This is about half the installation rate needed on average in the 2020s in the U.S. net-zero scenario, about one-quarter of the rate needed in the 2030s, and a still smaller fraction of the rate needed in the 2040s. This challenge is made all the more interesting by the fact that the U.S. currently imports the majority of it solar cells, and mostly from China.

This solar/wind capacity expansion illustration highlights the fact that achieving decarbonization ambitions, whether in China or in the U.S., will require significant increases in both techno-economic and sociopolitical capabilities in both countries. Similar challenges apply to other key pillars of decarbonization. Differences in natural resource endowments, governance structures, societal norms, and other factors imply that some capabilities might be built more rapidly in one country than the other, but ultimately, both countries must build capability in multiple dimensions. From the techno-economic perspective, designing, siting, mobilizing investment capital, and building physical plant and infrastructure at the pace and scale needed will likely require changing how these practices are done today so as to dramatically speed them up. From the sociopolitical perspective, earning and sustaining public support and social license for the large changes entailed in pursuing deep decarbonization will be essential. This includes ensuring that communities dependent on fossil fuel industries for their livelihoods today have alternative economic opportunities as those industries shrink. Modeling and analysis to help understand the feasibility of accelerating the building of capabilities needed to achieve ambitions requires a mix of approaches including higher-resolution spatiotemporal analysis of physical assets than has been common, together with related investigations of manufacturing capabilities, raw material supply chains, investment decision-making processes, the needs and attitudes of local communities, and many others.

Such modeling and analysis should be designed to inform the development of new government policies across the economy that will be needed to drive change of the magnitude involved in transitioning to net-zero emissions. There are many possible policies that could be deployed, the effectiveness of which can vary substantially with local conditions. This points to the need for more granular and localized modeling and analysis to support policy formulation efforts. Design and assessment of specific policies are beyond the scope of our paper, but we note that it is important to track progress continuously and transparently toward decarbonization goals in order to assess the effectiveness of polices and make iterative adjustments. Modeling can play an important role in this respect. For example, tracking metrics could be organized around the six pillars discussed above. Table 2 gives some illustrative metrics; a more comprehensive list would include many others.

**Table 2:** A list of key benchmark indictors to assess the progress of realization in national decarbonization key pillars

Decarbonization pillar	Benchmark indicators
Energy efficiency and electrification	Primary energy use per GDP (energy intensity)
	Fraction of final energy that is electricity by sector and total
	Total and sector-specific industrial energy intensity and electrification rate, as supported by energy-use per product and recycling rate of materials
	Fraction of light-duty vehicle fleet that is battery-EV
	Fraction of residential space heating done with electric resistance/heat pumps
	Number of EV charging stations
Clean electricity	Annual capacity/generation of solar and wind electricity
	Annual capacity/generation of nuclear power
	Annual capacity/generation of fossil fuel with CCS
	Annual capacity/generation of unabated fossil electricity
	Annual electricity transmission/distribution capacity
	Annual capacity/generation of low-carbon flexible resources, e.g., combustion turbines and batteries
Clean fuels	Annual supply of sustainable biofuels
	Annual production of low/zero/negative-carbon H_2_
	Annual consumption of gasoline, diesel, and jet fuel
CO_2_ capture and storage	Annual CO_2_ capture from power sector
	Annual CO_2_ capture from other sectors
	Annual CO_2_ utilization in various sectors
	Annual CO_2_ geological sequestration
Reduced non-CO_2_ emission	Annual CH_4_ emissions by source
	Annual fluorocarbon emissions by source
	Annual N_2_O emissions by source
Land sink	Annual deforested and reforested wood volumes
	Annual agricultural CO_2_ absorption

The metrics in Table 2 are largely lagging indicators that measure progress in emissions reductions (or lack thereof). Given the long lead times of decarbonization projects as they go through feasibility analysis, permitting, financing, and construction, it would be beneficial to also include some leading indicators. On the supply side, such leading indicators could include the number of projects in different stages of development, for example, proposals in planning, proposals under consideration by regulatory authorities, and projects under construction. On the demand side, leading indicators might include orders of new electric vehicles not yet delivered. Similarly, it would be beneficial to track metrics further upstream in the energy sector value chain, for example, manufacturing capacity for renewable energy and electric vehicles, land area of bioenergy crops under cultivation, investments in CO_2_ storage characterization, and new mining projects to supply critical minerals for the energy transition.

## Discussion

The above comparative analysis of the China and U.S. studies provides some insights on how energy-systems models can be improved to better inform ESGO processes. The two modeling studies adopted contrasting approaches, each having strengths and weaknesses. The China study used a mixed “bottom-up” and “top-down” approach. For example, it used expert judgment to establish overall economy-wide CO_2_ emissions allowable by sector, but some sectors (e.g., fuels and industrial materials) were not modeled in a detailed, integrated fashion. This resulted in some skewed treatment of sectors. For example, almost all CO_2_ capture for the country is found in the electricity sector, which may not be the most reasonable approach from a national, multisector perspective. Meanwhile, the U.S. study involved “macro-energy system” modeling to construct different technological pathways to meet a 2050 net-zero emissions target, allowing for highly optimized cross-sectoral interactions, e.g., between electricity and fuel sectors. The U.S. modeling showed that cross-sector interactions, especially late in the transition, make key contributions to achieving decarbonization goals, but such interactions represent idealized behaviors with little historical precedent in the U.S., which raises questions about their feasibility.

The China study involved experts making collective judgments, informed by various independent sub-sector modeling work, as to which pathways/timelines are the most feasible or realizable, given technical, social, environmental, and political conditions of the country. The U.S. study modeled and mapped, at high spatial and sectoral resolution, the equipment and infrastructure deployments that would allow the country to reach net-zero emissions by 2050 under five technologically different pathways. In effect, the China study applied expert judgment to project decarbonization capabilities and then set ambitions accordingly, while the U.S. study set the ambition and then used an idealized whole energy-system model to quantify the capabilities needed to achieve that ambition, without the kind of expert judgment utilized in the China study as to whether the country has or can develop those capabilities.

Although the approaches of the two studies are different, both provide insights in terms of ambition, capabilities, and realization that can inform complementary analyses and policy formulation efforts within an ESGO framework that are essential for making decarbonization progress. Iterative application of the ESGO framework will also lead to improved models, and we expect that the two modeling approaches would converge over time. Both modeling studies have helped inform the formulation of governance measures aimed at driving the transition in each country. Quantitative metrics derived from the studies, such as those discussed in the previous section, provide a means going forward for assessments of the extent to which a country's capabilities are being deployed to support the transition. In the case of China, these assessments can provide input to iterations of the study, e.g., by informing updated expert judgments about China's decarbonization capabilities and identifying where higher-resolution and/or integrated cross-sectoral modeling would be valuable. In the case of the U.S., the assessments can be used as input to model iterations that provide updated assessments of the capabilities needed to achieve the ambition, which may provoke a reconsideration of the ambition level if the gap between needed capabilities and observed deployments of capabilities is judged to be too large. In light of such considerations, it may be concluded that applying the ESGO framework iteratively could allow more feasible pathways to be identified.

Finally, the complementarity of research methods and results between the two countries also fully shows the space for collaboration, shared learning, and further improvement of methods. For example, China has strengthened the breadth and depth of expert intervention to reinforce its impact on governmental policies, while the United States has paid more attention to the quantitative simulation and demonstration of new technologies such as hydrogen and CCS, especially the layout of infrastructure with high temporal and spatial resolution. This argues for greater international exchange and cooperation on decarbonization paths to accelerate the global decarbonization process, by resolving barriers affecting national ESGO systems, to accelerate the virtuous cycle of ambition, capability, and realization in the world.

## Conclusions

Greenhouse gas emission trajectories in most countries today consistently fall short of model-produced trajectories that achieve national GHG emissions reductions consistent with global ambitions embodied in the Paris Agreement. To a degree, this reflects the fact that contemporary energy-system modeling studies have not adequately factored in the techno-economic and sociopolitical capabilities needed to achieve the targeted emission reductions. New approaches to designing decarbonization pathways are needed for there to be a chance of accelerating the realization of mitigation ambitions, both in individual countries and for the world as a whole.

Toward that end, we have proposed here a framework, “Emissions-Sustainability-Governance-Operation” (ESGO), to facilitate translating mitigation ambitions into enabling policies that incentivize the investments needed to leverage local capabilities in realizing emissions reductions. The ESGO framework is applicable in any country. Details of how it is applied will differ from country to country depending on governance structures, organization of the energy system and related markets, and other factors. In all cases, however, it is important that application of the framework is informed by well-designed energy-system modeling studies, which produce insights that connect ambition, capability, and realization. Models can facilitate calibrating progress toward decarbonization goals, recalibrating enabling policies when needed to best leverage capabilities, and thereby progress the decarbonization transition as rapidly as possible.

The modeling studies for China and the U.S. discussed in this paper have complimentary features that are important characteristics of the kinds of modeling studies that can best inform application of the ESGO framework. A key feature of the China study is extensive consultation with leading experts to assess decarbonization capabilities sector-by-sector. A key feature of the U.S. study is the high spatial, temporal, and sectoral resolution for which decarbonization pathways were constructed. Modeling that combines these two key features can begin to enhance the feasibility of modeled energy system transition pathways. But realizing mitigation ambitions requires analyses and actions beyond energy-system modeling alone. The proposed ESGO framework, including improved energy-system modeling efforts, offers a common analytical structure and process that can be applied in different countries to further both national and international progress on decarbonization.

## Supplemental information

Document S1. Figures S1–S9

Data S1. Tabular data for Figures 2–5.

## Footnotes

Supplemental information can be found online at https://doi.org/10.1016/j.isci.2021.103695.
