# Supplementary material for: Realizing ambitions: A framework for iteratively assessing and communicating national decarbonization progress
Source: iScience. 2021 Dec 25;25(1):103695. doi: 10.1016/j.isci.2021.103695 (PMC8749441; doi:10.1016/j.isci.2021.103695)
Supplement: Document S1. Figures S1–S9 [file mmc1.pdf]

iScience, Volume *25*

Supplemental information

Realizing ambitions: A framework for iteratively assessing and communicating national decarbonization progress

Chuan Zhang, Honghua Yang, Yunlong Zhao, Linwei Ma, Eric D. Larson, and Chris Greig

1. Overview of China's Long-term Low-Carbon Development Strategy and Pathway study

The Institute of Climate Change and Sustainable Development (ICCSD) was founded after China's international announcement of its Nationally Determined Contribution (NDC) in 2016 to be an ambitious high-end "think and do" tank and a "torch bearer" to lead an acceleration of the process of tackling climate change in China and the world.[1] Mr. Xie Zhenhua, China's special representative on climate change, was appointed to lead the ICCSD. The CLLDSP study is a flagship project of the ICCSD to demonstrate and promote China's ambition on tackling climate change.[2] The project adopted a comprehensive methodology to maximize the combined understanding and insights offered through modeling, data analysis, and expert knowledge (see Figure S1). In addition to using holistic and sub-domain models for data integration, 15 workshops were held to promote cross-cutting analysis and interdisciplinary exchange among the participating institutions. This allowed the project to benefit from a combination of qualitative and quantitative analysis, and across the physical and social sciences. After three years of research and discussion, the abstract of project results was formally released on October 12, 2020, twenty days after China announced its 2060 carbon-neutrality ambition.

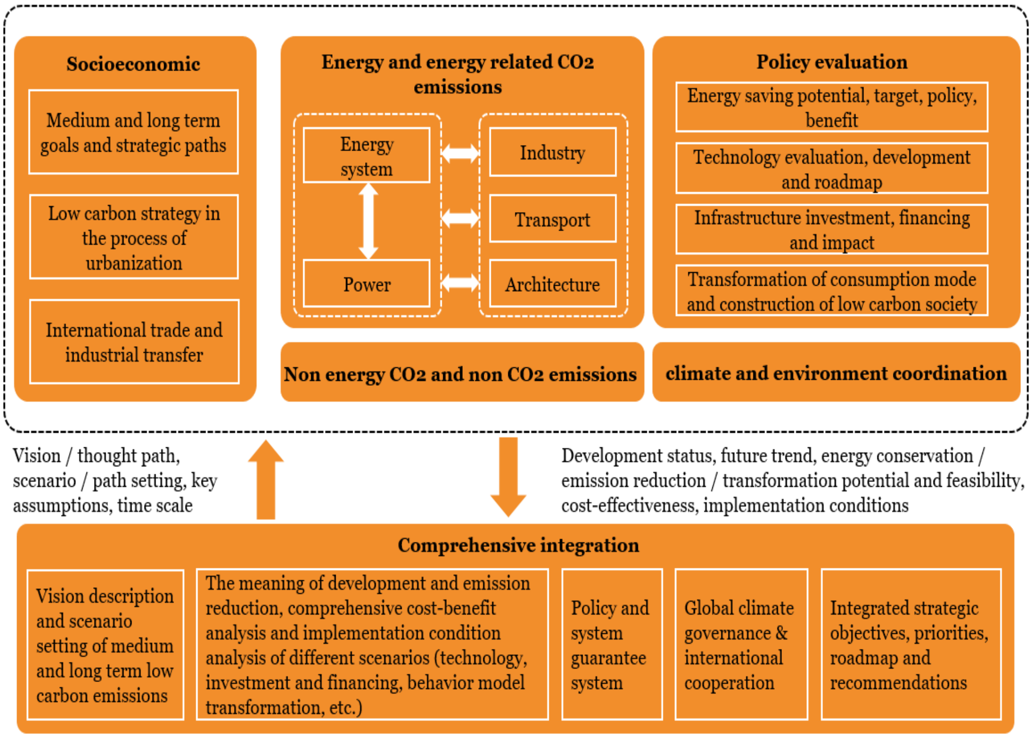

Figure S1. Structure of China's Long-term Low-Carbon Development Strategy and Pathway (CLLDSP) study.[2]

The aim of the CLLDSP was to develop pathways that achieve low emissions by 2050. Initially, four scenarios were identified (see Figure S2): 1) the Current policy (P) scenario meets China's 2030 NDC target, but assumes future policy measures are insufficient to achieve deep emissions reductions; 2) the Strengthened policy (SP) scenario meets the 2030 NDC target and bends the emissions curve further than (P) by 2050; 3) the 2℃ scenario substantially reduces emissions by 2050, consistent with meeting that goal of the Paris Agreement; and 4) the 1.5℃ scenario represents a pathway incorporating all possible efforts to achieve the lowest emissions by 2050, at least net-zero CO_2 emissions by 2050, and with this scenario net-zero GHG emissions would be realized before 2060.

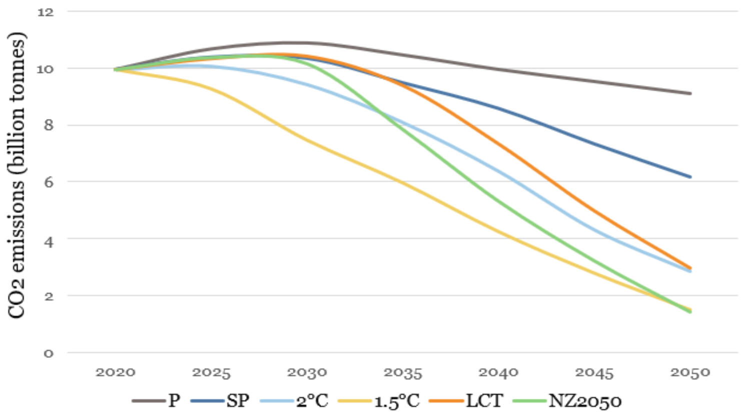

Figure S2. Energy-related CO_2 emissions of all investigated emission trajectories for CLLDSP study.

The ICCSD recommended the 2℃ and 1.5℃ scenarios for setting China's net-zero emissions ambitions, by suggesting only 3.94 and 1.33 billion tonnes GHG emissions in 2050, or 68% and 90% below 2020 levels, respectively. The latter scenario can realize net-zero CO_2 emissions by 2050 and then very likely net-zero GHG emissions by 2060. These results were derived by careful assessment of the trade-off between external and internal ambitions. China's *external ambition* is to actively tackle climate change as the biggest developing country, taking into consideration its export-oriented economy and growing international standing. China's *internal ambition*, which is influenced by China's governance system and increasing public concerns about the state of its natural environments, is to vigorously build an ecological civilization and a beautiful China. Net-zero GHG emissions by 2050 was not proposed, because China desires high economic growth rates appropriate for a country that is still developing and, considering its current dependence on coal and the huge scale of its energy-intensive industries, a more rapid reduction in emissions would require unacceptable sacrifice of economic growth and/or energy security. The ICCSD recommendations for 2050 targets thus reflect China's twin objectives of actively fulfilling the Paris agreement and achieving socialist modernization by 2050.

Beyond recommending the 2050 targets, further deliberations of the CLLDSP study team led to two additional scenarios that reflect the team's judgement that China currently does not have the *capabilities* for realizing emission reductions in the near term at the rates modeled in the 2℃ and 1.5℃ scenarios (Section 3 illustrates quantitatively some of the challenges). The team's consensus was that existing capabilities can only support a slowing of emissions growth, as in the SP scenario, until emissions peak no later than 2030. During the period to 2030 China would be able to build its capabilities to more rapidly reduce its emissions from around 2030 to meet the 2050 emission targets of the 2℃ or 1.5℃ scenarios, resulting in the "Long-term low-carbon transition (LCT)" and "Net-zero CO_2 emission by 2050 (NZ2050)" scenarios in Figure S2. This judgement was arrived at through organized assessments of required techno-economic capabilities, e.g., ability to deploy end-use energy efficiency and electrification, and of required socio-political capabilities, e.g., for institutional reform and policy design.

With the goal of realizing the ambitions reflected in the "Net-zero CO_2 emission by 2050" scenario, the CLLDSP study recommended a series of near, medium, and long-term goals and policy measures aimed at achieving these. The recently delivered 14th Five-Year Plan (FYP) (2021-2025) of China reflects the recommended near-term targets for reduced energy intensity (energy consumption per unit GDP) and carbon intensity (CO_2 emissions per unit GDP), albeit with some adjustments to account for the influence of the pandemic on the economy. Other suggestions made for the 14th FYP period included: mandating that key cities and energy-intensive industries reach peak CO_2 emissions and have ten-year plans for continued reductions; striving for coal consumption to peak or even start to decline; improving the design of the national carbon market and extending its sectoral coverage; and reducing methane and other non-CO_2 greenhouse gas emissions and establishing monitoring, reporting, and verification systems. Policy suggestions for realizing medium and long-term ambitions focus largely on the idea of strengthening institutional capabilities to address climate change.

2. Overview of US's Net-Zero America study

The Net-Zero America (NZA) study,[3] led by researchers at Princeton University and involving a large team of researchers across several other U.S. organizations, was motivated by the goal of providing actionable analysis to inform public and private decision making at federal, state, and local levels. The 2-year effort provides unprecedented sectoral, spatial, and temporal detail in describing multiple technological pathways that would result in the United States achieving net-zero greenhouse gas emissions by 2050. The analysis quantifies challenges and opportunities at state and sub-state levels, including those relating to land use, employment, air pollution-related health, capital mobilization, incumbent fossil fuel industries and new clean-energy industries. Since the study's release in December 2020, its uniquely high spatial resolution has attracted attention from federal and state policy makers, private industry, non-governmental organizations, and inspired similar national studies around the world. The study has also attracted extensive media attention.[4]

Using a suite of models (see Figure S3), the NZA analysis identified five distinct pathways by which the U.S. could reach net-zero emissions economy wide by 2050, factoring in assumptions about future non-CO_2 emissions and carbon uptake in soils and trees. The starting point for the modeling was an official US Department of Energy projection to 2050 of energy-service demands by region and sector throughout the economy. Market penetration rates of energy end-use technologies that meet the service demands were exogenously specified and resulted in a set of final energy demands to be delivered by the energy supply system. A linear-programming optimization model then chooses the mix of primary energy sources and conversion technologies that minimizes total energy system costs while meeting the final energy demands and achieving net-zero emissions by 2050. This results in descriptions of the energy and industrial system at a relatively coarse (14-region) geospatial resolution. Various modeling methods were applied to "downscale" those results to state, county, or finer spatial resolution.

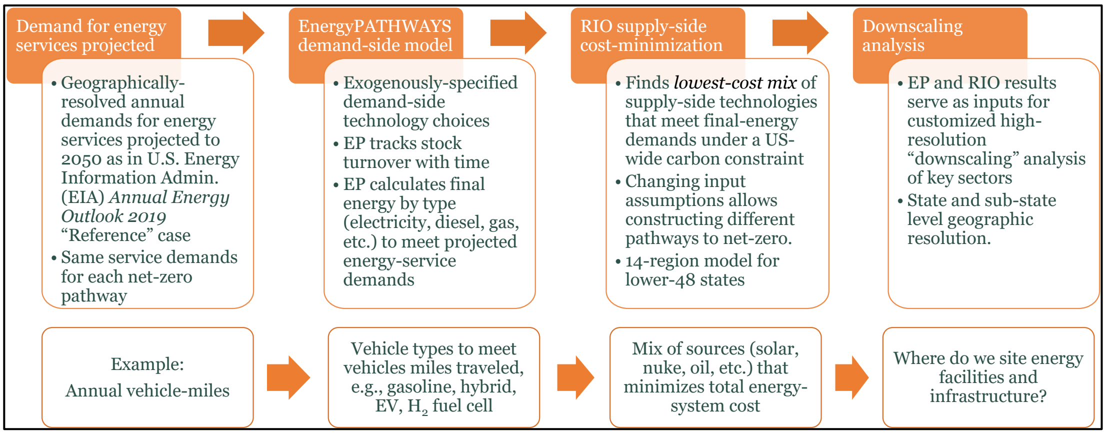

Figure S3. Modeling approach used in the Net-Zero America study.[3]

The NZA study aims to provide granular guidance on what actions are needed to realize a U.S. ambition of net-zero emissions by 2050, given the country's natural resource base. With that in mind, all five of the modeled NZA pathways meet an exogenously imposed linear reduction in emissions to net-zero in 2050, but with varying other constraints imposed to create five distinct technological pathways (Figure S4). REF refers to a reference "no new policies" scenario, in which there are no economy-wide emissions constraints applied in the modeling.

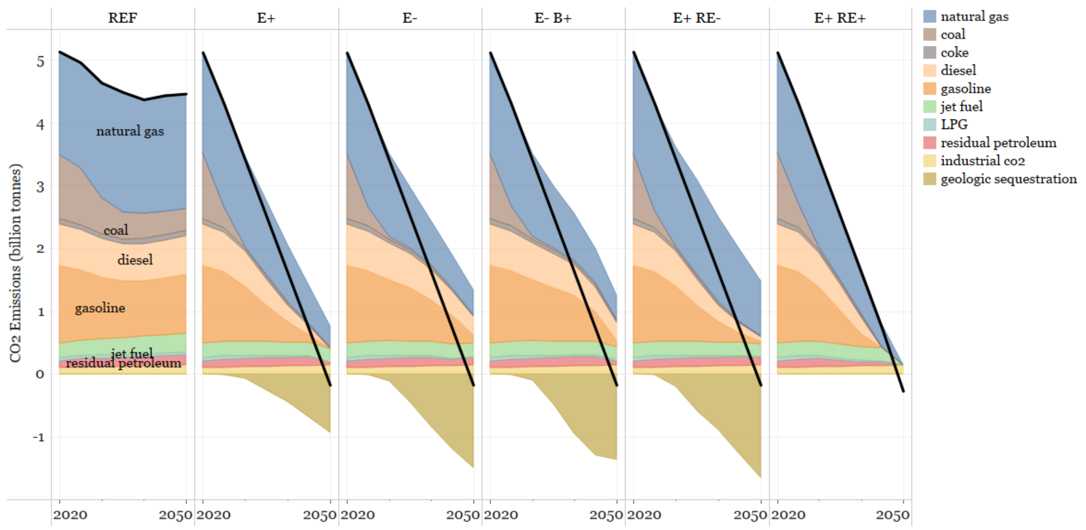

Figure S4. CO_2 emissions from the U.S. energy/industrial system for five pathways achieving net-zero emissions economy wide by 2050

1) in the E+ pathway, aggressive end-use electrification is assumed; energy-supply options are essentially unconstrained, except for a requirement that there be no change in land use for biomass energy supply compared with today's pattern of land use;

2) in E-, less aggressive end-use electrification is assumed, and energy-supply options are as in E+;

3) in E-B+, the less-aggressive end-use electrification is adopted, but an expanded biomass supply is allowed by conversion of some cropland or pasture to biomass energy production;

4) in E+RE-, aggressive end-use electrification is assumed, and the annual amount of wind and solar generating capacity that is allowed to be built is limited to near the highest single-year installation rate achieved previously in the U.S.;

5) in E+RE+, aggressive end-use electrification is adopted, and several supply-side constraints are applied, including eliminating fossil fuel use by 2050 and not allowing any new nuclear power construction or underground CO_2 sequestration.

The NZA study is neutral as to which of the pathways is "best", but by presenting highly granular depictions of required physical, institutional, and human resource efforts to reach net-zero along each pathway, the study aims to help focus the attention of decision makers and stakeholders on key challenges and impacts and trade-offs involved between pathways. Exogenous assumptions of non-CO_2 emissions and land carbon sinks are included in the modeling and result in target net emissions from the energy/industrial system in 2050 of negative 170 million t CO_2 (carbon storage in long-lived products is included in the modelling yet is not shown explicitly).

The ambition represented in the NZA study mirrors the ambitions of the current leadership of the U.S. government, which has pledged to reach net-zero emissions by 2050, with an interim goal of reducing emissions by at least 50% (relative to 2005) by 2030. The leadership's external ambition is reflected in its hosting of the Leader's Summit on Climate in April 2021.[5] Its internal ambition is reflected in a statement it released on that occasion, which states in part: "Climate change poses an existential threat but responding to this threat offers an opportunity to support good-paying, union jobs, strengthen America's working communities, protect public health, and advance environmental justice. Creating jobs and tackling climate change go hand in hand – empowering the U.S. to build more resilient infrastructure, expand access to clean air and drinking water, spur American technological innovations, and create good-paying, union jobs along the way." [6]

The NZA study demonstrates that the U.S. has sufficient capabilities, in terms of natural resource capital, to support a transition to net-zero emissions. On the other hand, the study

also demonstrates, as examples in the next section illustrate, that heightened capabilities to deploy human, social, manufactured, and financial capital must be established and expand throughout the full transition to 2050 at historically unprecedented rates in order to realize the net-zero ambition. With its highly granular analysis, the NZA study provides a blueprint for actions needed in the next decade to build the capabilities needed to put the U.S. on a path to realizing net-zero emissions by 2050. The study does not make specific policy recommendations for building capabilities, but instead provides quantitative benchmarks against which progress can be measured at socially and politically relevant geographic and sectoral resolutions.

3. Comparative results of the US-China study

To better understand and appreciate the differences between China's and United States' ambitions and capabilities for net-zero emissions, it is instructive to compare some results from the two studies. The studies differ in the approaches used, but both rely on the same six key pillars of decarbonization:

(1). End-use energy efficiency and fuel switching to electricity (electrification), especially in the transportation and buildings sectors.

(2). Clean (zero or negative carbon) electricity generation, including from variable renewables (wind and solar) and firm sources to balance the variable resources (biomass, nuclear, gas with CCS, storage), as well as expanded transmission to move remotely generated electricity to demand centers.

(3). Clean (zero or negative-carbon) liquid and gaseous fuels, including biofuels, hydrogen, and synthesized liquid hydrocarbons.

(4). CO_2 capture and utilization or storage (CCUS).

(5). Reduced emissions of methane and other non-CO_2 GHG gases.

(6). Enhanced uptake of atmospheric CO_2 in the biogeosphere (i.e., nature-based solutions), providing negative emissions.

3.1 Pillar 1: Improve energy productivity through efficiency and electrification

Ambitious improvements in energy-efficiency characterize both the US and China decarbonization pathways. The overall energy efficiency of an economy is reflected in its primary energy use per unit of gross domestic production, or its energy intensity. In the U.S. net-zero pathway, primary energy use falls by about 30%, from 98 EJ in 2020 to 70 EJ in 2050, while GDP is assumed to grow 1.8% per year, resulting in a decline in energy intensity by 60%, from 5.3 to 2.1 MJ/$GDP (S5a). In China's pathway, primary energy use continues to grow from 145 EJ in 2020 to a peak of 155 EJ in 2030 before declining back to 146 EJ in 2050. Meanwhile China's GDP is assumed to grow on average 4.2%/y from 2020 to 2050, resulting in energy intensity falling by 70%, from 10.3 in 2020 to 3.1 MJ/$GDP in 2050.

The declines in energy intensity result from assumed decreases in energy use per unit of energy-service delivered and shifts in economic structures toward higher value-added activities. These energy intensity targets result in primary energy use per-capita in the US falling to 185 GJ/person by 2050 (nearly half the 2020 level) and remaining nearly flat at 105 GJ/person in China.

Ambitious progress in electrification, and the intrinsic reductions in final-energy use per unit of energy service provided that can accompany it, is another key feature of the transition in both studies. The percentage of electricity in total final-energy use is approximately 20% in both China and the U.S. today. The U.S. net zero transition has this fraction increasing to 50% in 2050, and China sees an even bigger shift, with electricity reaching nearly 2/3 of final energy use by 2050 (Figure S5b). These shifts include increased electrification of industrial production and massive expansion of both heat-pump electric heating of buildings and battery-electric light-duty vehicles.

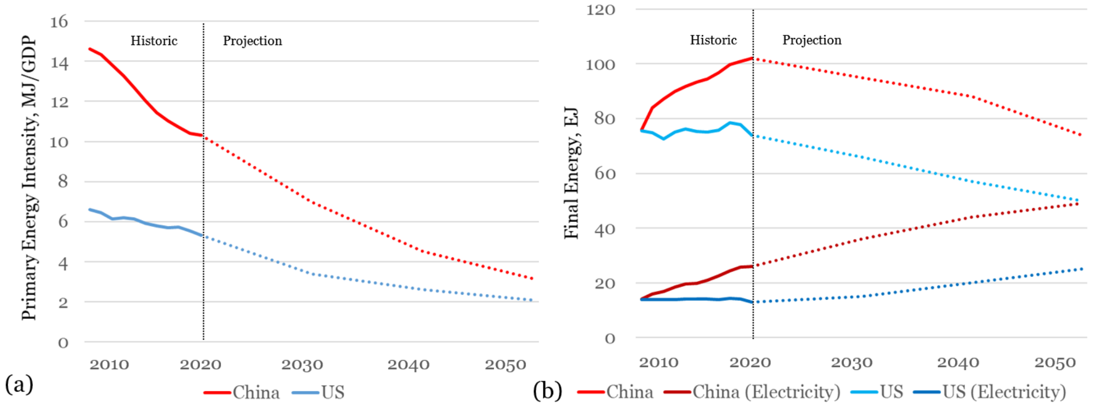

Figure S5. Historic and projected (a) primary-energy intensity (MJ per $GDP) and (b) final energy (total and electricity portion).

3.2 Pillar 2: Clean Electricity

Both the U.S. and China studies have ambitious goals for expanding electricity generation and the portion from carbon-free generation, both variable renewables (e.g., solar and wind) and clean firm generation (hydro, nuclear, biomass, geothermal, fossil with CO_2 capture). Total electricity demand doubles from 2020 to 2050 in both studies (Figure S6a), requiring 3 and 3.5 times as much generating capacity in China and the U.S., respectively (Figure S6b), operating with an average capacity factor lower than today due to the large wind and solar fractions. By 2050, the portion of solar/wind electricity generation reaches 60% for China and 80% in the U.S. Clean-firm generation plays a larger role in China than in the U.S: by 2050 nuclear, hydro, and coal with CCS in China account for 16%, 10%, and 6% of total generation, respectively (Figure S6b); in the US case, nuclear, hydro, and gas with CCS account for 5%, 3%, and 2% of total generation, respectively.

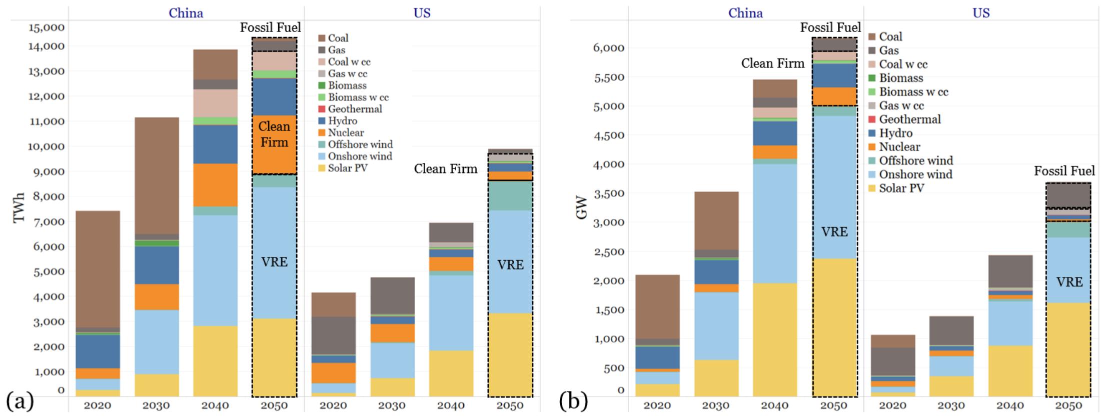

Figure S6. Electricity generation (a) and installed capacity (b) for U.S. and China decarbonization scenarios. The bars for 2050 also highlight the fractions that are variable renewable electricity (solar and wind), clean firm (hydro, nuclear, biomass, geothermal, fossil with CO_2 capture), and fossil fuels (without CO_2 capture).

Expansion rates for both variable renewables and clean-firm generation observed in the past in the U.S. and in China provide an indication of each country's current capabilities for future expansions. In 2020 China installed a record 72 GW of wind capacity and a near-record 48 GW of solar PV. This combined 120 GW of new capacity comes close to the average build rate in the 2020s in China's decarbonization scenario (Figure S7). An average of nearly double this rate would be needed in the 2030s. For the U.S., the record U.S. build year so far was also 2020, when 24 GW of wind and utility-scale solar PV capacity were installed, plus about 5 GW of rooftop solar. The average annual installation of wind and utility-scale solar PV capacity needed in the 2020s for the U.S. net-zero scenario is about double the amount installed in 2020, and the average installation rate doubles again for the 2030s and grows still more in the 2040s

(Figure S7). The U.S. study estimated the land area needed for solar and wind farms by 2050 would be nearly 600,000 km^2. For China, with 1.5 times the solar capacity of the U.S. in 2050 and 2.2 times the wind capacity, the estimated land required is 1.3 million km^2. While these are significant land areas, both China and the U.S. possess abundant land, so land availability perse, may not be constraining.

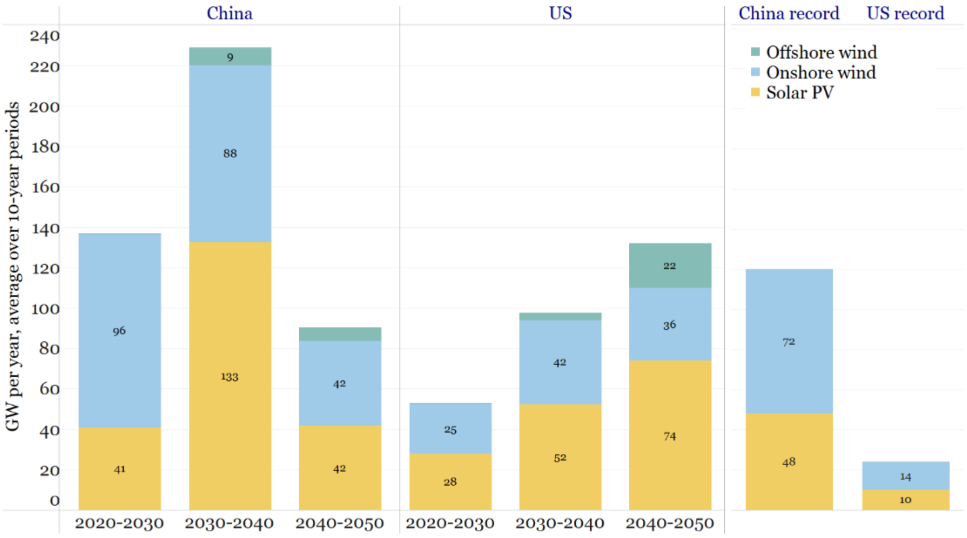

Figure S7. Average annual expansion (GW/y) each decade in solar and wind generating capacity in the China and U.S. decarbonization studies. The two right-most bars show the largest single-year expansions achieved in the past. For China, these occurred in 2020 for wind and in 2017 for solar, as for the U.S., both occurred in 2020.[7]

High voltage transmission capacity expansions would be needed to support such high levels of added wind and solar. In the U.S. case, a tripling of transmission capacity is needed by 2050. It is informative to note that the current US transmission system took over 100 years to build; meeting the net-zero ambition would require building again as much transmission capacity in just 15 years and then doing the same again in the following 15 years. In the China case, a five-folder increase is needed to support interregional electricity transmission, namely from around 240GW today to 1400GW in 2050.

New nuclear power capacity is an important clean-firm generation source for China’s 1.5°C scenario but plays only a small role in the US net-zero pathways (except when wind and solar capacity expansion rate fails to grow e.g., the E+RE- scenario). By the 2040s, capacity additions average about 10 GW per year in China. For comparison, China brought an average of nearly 5 GW per year of new nuclear capacity online from 2014 to 2020, with a peak of 8 GW in 2018.[8] Fossil fuel power with CCS is an important player in both the China and US scenarios. By 2050 in China, 157 GW of coal with CCS are installed whereas in the US, 87 GW of gas with CCS are online. Neither scenario relies on fossil fuel with CCS before 2030, which allows time for maturation of CCS technology.

These comparisons indicate that techno-economic capabilities for expanding clean electricity supplies must grow rapidly in both China and the U.S. if the modeled decarbonization trajectories are to be achieved. The comparison of annual energy-infrastructure expansion rates with record past expansion rates suggests that associated socio-political capabilities for implementing such rapid changes may also need to enlarged in both countries.

3.3 Pillar 3: Clean fuels

Despite the rapid electrification of energy end uses in both the U.S. and China low-carbon scenarios, there are still end-use demands for fuels and feedstocks of around 25 EJ in 2050 in both scenarios. The U.S. study details how these fuel demands are satisfied. About 65% are met using fossil fuels whose emissions are offset by negative emissions elsewhere in the economy. Hydrogen provides about 20%, and the rest are in the form of lignocellulosic biofuels and fuels synthesized from hydrogen and captured CO_2. Total hydrogen production for end-use

or as input to fuels synthesis is 58 million t/y, or nearly six times current U.S. hydrogen production. Unlike today, where hydrogen is made exclusively by steam methane reforming, hydrogen in the 2050 scenario comes from several processes, none of which are commercially deployed today: autothermal methane reforming with CO2 capture and storage (4 million t/y), electrolysis using clean electricity (21 million t/y) and biomass gasification (34 million t/y). The latter, which consumes about ¾ of the 12 EJ of biomass used in 2050, includes CO_2 capture and storage and so provides negative-emission hydrogen.

The fuel sector in the China study was not modeled in detail, but as in the U.S., hydrogen is expected to play an important role. The 1.5oC scenario for China includes a demand for hydrogen in 2050 of close to 60 million t/y, accounting for about 10% of end-use energy. Hydrogen is produced in China today from coal or petroleum-refining residuals. In the 1.5oC scenario, hydrogen from non-fossil energy sources and coal gasification with CO_2 capture and storage are expected to play important roles. The option of biomass gasification for hydrogen production is not prominent in the China study, though the amount of biomass resources projected to be available for energy uses in 2050 (9 to 15 EJ/y) is comparable to the biomass level in the U.S. study. Biomass is used primarily for power generation with CO_2 capture and storage in the China study so that the negative emissions can offset remaining carbon emissions in the electricity sector, and also considered to be an important low-carbon option of domestic fuel for buildings.

China and the U.S. face some similar challenges for the transition to clean fuels. For example, while the key modeled hydrogen production technologies are available today, none are commercially deployed at scale due to cost and other hurdles. Also, infrastructure to support hydrogen end-uses is virtually non-existent today, outside of a few selected industries. In the case of biomass, whether used for fuels or power, the extent of feedstock collection and processing infrastructure needed to facilitate wide use of biomass is very limited today, and technologies for CO_2 capture on biomass conversion plants are not well developed.

3.4 Pillar 4: CO_2 Capture and Storage

Ambitious CO_2 capture and storage (CCS) rates are assumed in both the China and U.S. studies with close to 1 billion tonnes per year being captured and stored by 2050 (Figure S8). At a nominal storage-reservoir pressure, this volume of CO_2 is equivalent to 1.3 times current U.S. crude oil production (~4 billion barrels per year). Most (72%) of the CO_2 capture in the U.S. study occurs at hydrogen and fuels production facilities, with the rest split between power sector and cement plants. In the China study, coal continues to play an important role in power generation, necessitating that most of the CO_2 capture occurs in the power sector. Biomass power generation with CCS is important in the China study to provide negative emissions to offset remaining electricity-sector carbon emissions.

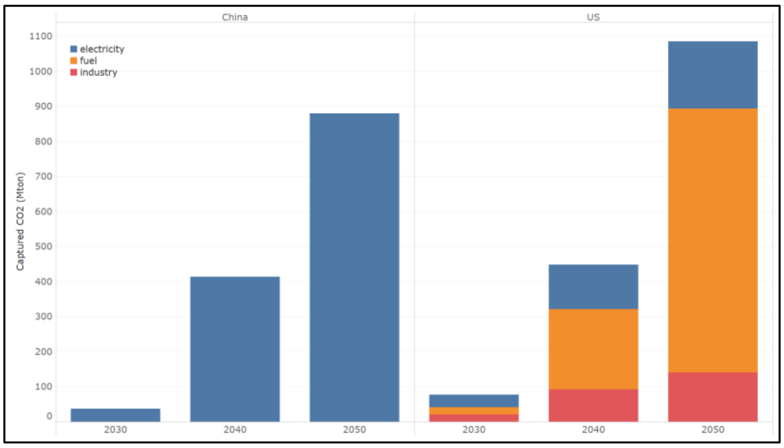

Figure S8. Source of CO_2 capture in the two studies.

The capability to achieve such ambitious rates of CCS in either country hinges on:

CO_2 capture equipment becoming commercially established and engineering firms having the

capabilities to design and build capture installations at the pace required. Several technically successful commercial-scale capture projects in the U.S. and China are the starting point for this development.[9]

Exploration, appraisal and development of underground storage formations. In the U.S., the Gulf Coast region is considered to have high suitability for storage, and there are additional suitable storage basins elsewhere in the country. The U.S. is thus relatively well endowed with storage resources, but further work is needed to convert these into storage resources into storage reserves. There has been less work done in China to understand the potential suitability of underground formations for CO_2 storage.

Establishing the industrial capabilities for CO_2 transport and injection. This will require skills and equipment similar to those for oil and gas production and transport. U.S. capabilities in this regard are vast and well established by comparison with those in China.

3.5 Pillar 5: Reducing non-CO_2 greenhouse gas emissions

The US net-zero modeling assumed as an input a level of mitigation from 2020 to 2050 consistent with recent analysis from the U.S. Environmental Protection Agency (EPA), adjusted for greater reductions in coal, oil and gas use than assumed by EPA. Without mitigation efforts, non-CO_2 emissions grow gradually to 1.45 $GtCO_2e$ by 2050. With abatement efforts, non-CO_2 emissions are reduced to about 1 $GtCO_2e$/y by 2050 (Figure S9a), with most of the remaining non-CO_2 emissions attributed to agricultural activities for which deeper emissions reductions are much more costly or not yet available.

Currently, China's NDC targets give no consideration to non-CO_2 emissions. But in the future, the emission reduction of non-CO_2 needs to arouse widespread attention and be included in the NDC targets. In China, non-CO_2 emissions were 2.17 billion tCO_2e in 2015, need to peak by 2020, at about 2.38 billion tCO_2e/y, and drop to 1.2 billion tCO_2e/y by 2050, or about half of the peak level.[10] Non-CO_2 emission reduction should reach 590 Mt CO_2e/y and 680 Mt CO_2e/y in 2030s and 2040s, respectively.

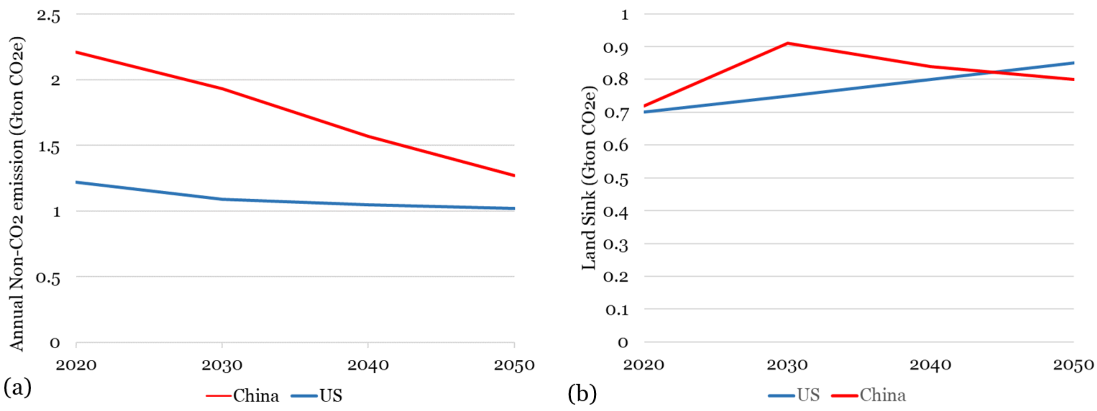

Figure S9. (a) Annual non-CO_2 greenhouse gas emissions for the US and China, (b) Annual land based greenhouse gas sink for US and China.

Without major technological innovation, non-CO_2 cannot achieve net-zero emissions by 2050, even when all optional emission reduction technologies are implemented, and the cost of deep emission reduction is very expensive. Therefore, the emission reduction of non-CO_2 needs to seize the early opportunities. The emission reductions of non-CO_2 by 2030 will mainly come from the coal mining process, while reductions from 2030 to 2050 will mainly come from the additional efforts of coal mining, animal enteric fermentation and solid waste treatment processes.

3.6 Pillar 6: Land sinks

Land carbon sinks (i.e., annual removal of carbon from the air and permanent storage in soil or trees) are critical for net-zero emission scenarios, because they offset positive greenhouse gas emissions from elsewhere in the economy. In U.S. modeling study, land sinks avoid the costliest

measures for CO_2 emissions reductions in the energy/industrial system.

There is uncertainty about what the magnitude of the U.S. land sink is today, but 0.7 $GtCO_2$/y is thought to be a reasonable estimate. Without efforts to enhance the natural land sink, it is projected to decline to 0.3 $GtCO_2$/y by 2050. Significant modification of agricultural and forestry practices, if widely adopted, can help maintain and enhance the land sink. Geographically resolved analysis for the U.S. estimates a technical potential for enhanced land sinks by 2050 of up to 0.2 $GtCO_2$/y in agriculture and from 0.5 to 1.5 $GtCO_2$/y in forestry. The U.S. net-zero modeling study assumed the land sink grows to 0.85 $GtCO_2$/y by 2050 (Figure S9b).

A similar level of land sink by 2050 (about 0.8 $GtCO_2$/y) is assumed for the China 1.5oC scenarios (Figure S9b). Research suggests that wetland and grassland carbon sinks will hold steady at 0.2 $GtCO_2$/year through 2050, and so the forest will become the focus of land carbon sink enhancement. Considering that the forest biomass is closely related to its growth stage, the accumulation of carbon in middle-aged forests is the fastest, while in mature forests/over-mature forests, the carbon absorption and release are basically balanced. Before 2030, it is necessary to accelerate the increase of afforestation area, strengthen the management of young and middle-aged forests, and strengthen the exploitation of mature forests, so that the carbon sink level will exceed 0.9 $GtCO_2$ by 2030. In the middle and late stages, on the basis of continuing to increase the afforestation area, select good tree species, improve the forest age structure, and maintain the carbon sinks at a relatively stable level. To achieve the 1.5℃ target, by 2050, the carbon sink capacity needs to be increased by 1.7 times, reaching 0.58 $GtCO_2$/y, which means that average annual growth rate of the forest stock needs to be 2.16%/y. According to historical data, China's forest stock volume was 11.27 billion m3 in 1995, and 17.56 billion m^3 in 2015, which was increased by 2.24%/y in 20 years.[11,12] China has the capability to achieve the goal of land sink construction.

References

1. Xinhua (2016). China signs Paris Agreement on climate change. http://www.chinadaily.com.cn/china/2016-04/23/content_24796558.htm.

2. He, J., Li, Z., Zhang, X., Wang, H., Dong, W., Chang, S., Ou, X., Guo, S., Tian, Z., Gu, A., et al. (2020). Comprehensive Report of China's Long-term Low-Carbon Development Strategies and Pathways. Chinese Journal of Population, Resources and Environment *18*, 263-295. 10.1016/j.cjpre.2021.04.004.

3. Larson, E., Greig, C., Jenkins, J., Mayfield, E., Pascale, A., Zhang, C., Drossman, J., Williams, R., Pacala, S., Socolow, R., et al. (2021). Net-zero America: potential pathways, infrastructure, and impacts. Final report (Princeton University). https://netzeroamerica.princeton.edu/the-report.

4. Seltzer M. (2020). Big but affordable effort needed for America to reach net-zero emissions by 2050, Princeton study shows. https://phys.org/news/2020-12-big-effort-america-net-zero-emissions.html.

5. United States Department of State (2021). Leaders Summit on Climate. https://www.state.gov/leaders-summit-on-climate/.

6. The White House (2021). FACT SHEET: President Biden Sets 2030 Greenhouse Gas Pollution Reduction Target Aimed at Creating Good-Paying Union Jobs and Securing U.S. Leadership on Clean Energy Technologies.

7. International Renewable Energy Agency (2021). Renewable Capacity Statistics 2021. https://www.irena.org/publications/2021/March/Renewable-Capacity-Statistics-2021.

8. World Nuclear Association (2021). Nuclear Power in China. https://world-nuclear.org/information-library/country-profiles/countries-a-f/china-nuclear-power.aspx.

9. Global CCS Institute (2021). CCS Facilities Database. https://co2re.co/FacilityData.

10. Ministry of Ecology and Environment of China (2016). The first two-year update report on climate change in the People's Republic of China. http://www.mee.gov.cn/ywgz/ydqhbh/wsqtkz/201904/P020190419522735276116.pdf.

11. National Bureau of Statistics of China (2000). China Statistical Yearbook 2000. http://www.stats.gov.cn/tjsj/ndsj/zgnj/2000/A03c.htm.

12. National Bureau of Statistics of China (2019). China Statistical Yearbook 2019. http://www.stats.gov.cn/tjsj/ndsj/2019/indexch.htm.
